# Increased rate of elective cesarean delivery following assisted reproductive technology: A letter to the editor

**DOI:** 10.18502/ijrm.v18i2.6425

**Published:** 2020-02-27

##  Dear Editor

Concerns have been raised over the outcome of pregnancies achieved via assisted reproductive techniques (ART). Despite their success, they may have unexpected effects on the health and quality of life of these patients and their children (1). The complete effect of ART on the process of pregnancy has not yet been entirely elucidated. In accordance with a report, one adverse effect of ART may be a higher risk of cesarean delivery for these women than in women who conceive spontaneously (2). A meta-analysis revealed that the use of ART, even in singleton pregnancies, increases the risk of adverse pregnancy outcomes, including cesarean delivery. In this study, the risk of cesarean delivery increased by 58% in singleton pregnancies with ART compared to normal pregnancies (3). In an Australian study, the rate of cesarean delivery with a singleton pregnancy following intracytoplasmic sperm injection (ICSI) and in vitro fertilization was reported to be approximately 50% (4). Other studies have noted an increased rate of elective cesarean delivery among primiparous women with a singleton pregnancy who conceived following ART, after adjusting for sociodemographic characteristics and pregnancy complications (2, 5).

In pregnancies following ART, both the women and their obstetricians are usually reluctant to accept the risks of a vaginal delivery and choose cesarean section (4). Therefore, cesarean section without indication, and at the request of the mother or obstetrician, increases in cases of pregnancy after infertility. In a Belgian study, one in five obstetricians agreed with performing a cesarean section after ICSI at the request of older nulliparous mothers (2). In a study in Taiwan, the planned cesarean rate was nearly threefold in women who received infertility treatments. The increased risk for elective cesarean delivery in singleton pregnancies following infertility treatment is not justifiable by advanced maternal age or a higher rate of morbidities during pregnancy. Approximately two-thirds of women who had planned cesarean birth chose a time for a cesarean section before their due date (5). However, presently, no recommendations are available from the American College of Obstetricians and Gynecologists (ACOG) concerning the mode of delivery for ART pregnancies (6). Counseling for women who become pregnant following infertility treatments is imperative to reduce unnecessary cesarean deliveries (5). Regardless of how stressful these pregnancies can be (2), the decision regarding

the mode of delivery should only be evidence-based (4). Vaginal birth should be encouraged in low-risk pregnancies. Midwife-only care under the supervision of an obstetrician can be appropriate for many of these women (7). This would also decrease the economic expenditure on these pregnancies (2).

Although the rate of cesarean delivery has increased in Iran (8), there are no specific data regarding cesarean delivery after ART. The choice of cesarean delivery following ART has become common as the management of these pregnancies is considered to be high risk. The actual medical condition of these women should be considered in determining the mode of delivery. Further research is required on the rate and indications for cesarean delivery following ART in Iran. Increased knowledge in this area can help healthcare professionals provide support for women who conceive via ART to make an informed decision regarding their mode of delivery.

**Fahimeh Ranjbar Ph.D., Maryam Gharacheh Ph.D.**

Nursing Care Research Center, School of Nursing and Midwifery, Iran University of Medical Sciences, Tehran, Iran.

## References

[1] Macaluso Maurizio, Wright-Schnapp Tracie J., Chandra Anjani, Johnson Robert, Satterwhite Catherine L., Pulver Amy, Berman Stuart M., Wang Richard Y., Farr Sherry L., Pollack Lori A. (2010). A public health focus on infertility prevention, detection, and management. Fertility and Sterility.

[2] Gillet E., Martens E., Martens G., Cammu H. (2011). Prelabour Caesarean Section following IVF/ICSI in Older-Term Nulliparous Women: Too Precious to Push?. Journal of Pregnancy.

[3] Qin Jiabi B., Wang Hua, Sheng Xiaoqi, Xie Qiong, Gao Shiyou (2016). Assisted reproductive technology and risk of adverse obstetric outcomes in dichorionic twin pregnancies: a systematic review and meta-analysis. Fertility and Sterility.

[4] Sullivan Elizabeth A., Chapman Michael G., Wang Yueping A., Adamson G. David (2010). Population-Based Study of Cesarean Section After In Vitro Fertilization in Australia. Birth.

[5] Chien Li-Yin, Lee Yu-Hsiang, Lin Yu-Hung, Tai Chen-Jei (2015). Women who conceived with infertility treatment were more likely to receive planned cesarean deliveries in Taiwan. Human Fertility.

[6] College Of Obstetricians American, Gynecologists

[7] Lynley A, Payne D, Dan L (2013). Midwifery and Assisted Reproductive Technologies. New Zealand College of Midwives Journal.

[8] S Azami-Aghdash, M Ghojazadeh, N Dehdilani, M Mohammadi, R Asl Amin Abad

